# Modulation of gastrointestinal vagal neurocircuits by hyperglycemia

**DOI:** 10.3389/fnins.2013.00217

**Published:** 2013-11-26

**Authors:** Kirsteen N. Browning

**Affiliations:** Department of Neural and Behavioral Sciences, Penn State College of MedicineHershey, PA, USA

**Keywords:** vagus, vagal reflex, afferent, 5-HT, gastric motility

## Abstract

Glucose sensing within autonomic neurocircuits is critical for the effective integration and regulation of a variety of physiological homeostatic functions including the co-ordination of vagally-mediated reflexes regulating gastrointestinal (GI) functions. Glucose regulates GI functions via actions at multiple sites of action, from modulating the activity of enteric neurons, endocrine cells, and glucose transporters within the intestine, to regulating the activity and responsiveness of the peripheral terminals, cell bodies and central terminals of vagal sensory neurons, to modifying both the activity and synaptic responsiveness of central brainstem neurons. Unsurprisingly, significant impairment in GI functions occurs in pathophysiological states where glucose levels are dysregulated, such as diabetes. A substantial obstacle to the development of new therapies to modify the disease, rather than treat the symptoms, are the gaps in our understanding of the mechanisms by which glucose modulates GI functions, particularly vagally-mediated responses and a more complete understanding of disease-related plasticity within these neurocircuits may open new avenues and targets for research.

## Vagal reflex control of gastrointestinal functions

Autonomic neurocircuits are vitally important in the integration of homeostatic functions including the co-ordination of vago-vagal reflexes regulating gastric motility and emptying, nutrient absorption and satiety signaling. Data from several laboratories, including our own, have demonstrated that a wide variety of gastrointestinal (GI) neurohormones, and neurotransmitters act both centrally and peripherally to modulate vagal neurocircuits regulating GI functions (Dockray, 2004, 2009; Travagli et al., 2006).

Sensory information (mechanical, chemical, osmotic) from the GI tract is transduced and transmitted centrally via the afferent vagus nerve, the cell bodies of which lie in the paired nodose ganglia. While there does not appear to be strict somatotopic organization of neurons within the nodose ganglia there is a trend toward a rostro-caudal viscerotopy where neurons innervating the esophagus are located rostrally while neurons innervating the stomach are located more caudally (Zhuo et al., 1997). The central terminals of these sensory neurons enter the brainstem via the tractus solitarius (TS) and terminate on neurons of the nucleus of the tractus solitarius (NTS) using predominantly glutamate as a neurotransmitter (Andresen and Yang, 1990; Andresen and Kunze, 1994; Baptista et al., 2005). Unlike the nodose ganglion, neurons within the NTS are organized in a viscerotopic manner; activation of gastric vagal afferents, for example, activates neurons within the subnucleus gelatinosus of the NTS, while the subnucleus centralis receives information relating to the sensory control of swallowing (Altschuler et al., 1989, 1991; Barraco et al., 1992; Broussard and Altschuler, 2000). NTS neurons are heterogeneous with respect to their biophysical, neurochemical, and pharmacological properties (Bailey et al., 2002, 2006; Baptista et al., 2005; Browning et al., 2011) which contribute to their integration of this vast volume of sensory afferent information with metabolic and hormonal signals as well as neural inputs from brainstem and other CNS nuclei involved the regulation of autonomic functions. Once assimilated and integrated, NTS neurons relay this information to the adjacent dorsal motor nucleus of the vagus (DMV) which contains the preganglionic parasympathetic motoneurons which provide the output response back to the upper GI tract via the efferent vagus nerve.

In contrast to neurons within the NTS, DMV neurons are not organized viscerotopically but rather in columns or spindles that span the entire rostro-caudal extent of the nucleus related to each of the five subdiaphragmatic vagal branches that innervate the viscera (Shapiro and Miselis, 1985; Jarvinen and Powley, 1999; Travagli et al., 2006). While DMV neurons are also heterogeneous with respect to their biophysical, neurochemical and pharmacological properties (Fox and Powley, 1992; Browning et al., 1999, 2005; Jarvinen and Powley, 1999; Martinez de la Pena y Valenzuela et al., 2004; Babic et al., 2011), as preganglionic parasympathetic neurons they are all *a priori* cholinergic and activate postganglionic neurons within the target organ of interest via release of acetylcholine to activate nicotinic receptors. Postganglionic neurons within the upper GI tract form two distinct pathways to control gastric functions; an excitatory pathway that increases gastric tone, motility, and secretion via activation of muscarinic cholinergic receptors, and an inhibitory pathway that decreases gastric functions via release of non-adrenergic non-cholinergic (NANC) neurotransmitters, principally nitric oxide and vasoactive intestinal polypeptide. Gastric relaxation, therefore, can be achieved by either inhibiting the tonically active cholinergic pathway or by activating the inhibitory NANC pathway (Travagli et al., 2006).

## Effects of glucose on gastrointestinal functions

Effective glucose sensing is critical for the efficient integration and regulation of a wide variety of physiological functions including the optimal regulation of glycemic levels. One of the most dramatic variations in physiological conditions occurs in response to meal ingestion when blood glucose levels increase dramatically. Glucose exerts profound vagally-mediated effects upon gastric motility and emptying, in part to stabilize excessive fluctuations in blood glucose levels following meal ingestion (MacGregor et al., 1976; Horowitz and Fraser, 1994; Ferreira et al., 2001; Rayner et al., 2001; Ishiguchi et al., 2002; Shi et al., 2003; Zhou et al., 2008). An increase in gastric motility in response to hypoglycemia accelerates nutrient delivery to the intestine allowing increased absorption and re-establishes plasma glucose levels whereas a hyperglycemia-induced decrease in gastric motility delays gastric emptying and reduces further glucose absorption preventing potentially prolonged, and damaging, elevations in glycemic levels.

Glucose is known to directly alter the activity of enteric nervous system neurons; intraintestinal infusions of glucose not only activates predominantly sensory neurons in the myenteric and submucosal plexuses of the upper small intestine (Liu et al., 1999; Sayegh et al., 2004; Vincent et al., 2011), it also appears to modulate the response of enteric neurons to other GI neurohormones such as cholecystokinin and serotonin (Roosen et al., 2012). Glucose appears to decrease gastric motility and delays gastric emptying primarily via indirect (paracrine) mechanisms of action, however. Glucose within the lumen of the intestine induces the release of neurohormones from enteroendocrine cells including releasing 5-HT from enterochromaffin cells within the proximal intestine as well as GLP-1 from L-cells in the distal intestine. These released neurohormones activate receptors (5-HT3 and GLP-1 receptors, respectively) on peripheral GI vagal afferent fiber terminals and the resulting excitatory signals are relayed centrally (Raybould, 1998, 1999, 2002; Glatzle et al., 2002; Raybould et al., 2003; Vincent et al., 2011). These sensory signals activate second order neurons within the NTS and, following integration, the subsequent vagal motor response induces gastric relaxation and delayed emptying (Zittel et al., 1994; Ferreira et al., 2001; Raybould et al., 2003; Zhou et al., 2008; Hayes et al., 2010; Vincent et al., 2011).

The vagal efferent pathway responsible for this glucose-induced gastric inhibition is somewhat controversial, however. Studies in rats have demonstrated that, within the brainstem, increasing extracellular glucose levels decreases gastric motility via inhibition of the excitatory cholinergic pathway rather than activation of the inhibitory NANC pathway (Ferreira et al., 2001; Shi et al., 2005) whereas other studies have suggested that the gastric relaxation induced following peripheral hyperglycemia was abolished by nitric oxide and VIP antagonists, suggesting that activation of the inhibitory NANC pathway was involved (Zhou et al., 2008). While differences in experimental protocols may account for some of these differences, it is unlikely to explain fully such divergent results. It is possible that different vagal efferent pathways are engaged by peripheral vs. central glucose, although this remains to be elucidated.

## Effects of glucose on vagal afferent neurons

Once absorbed, however, glucose enters the bloodstream from where it continues to exert profound effects upon vagal neurocircuits controlling GI functions. While glucose increases the firing rate of vagal afferent fibers innervating the GI tract (Mei, 1978, 1985), it has also been known for some time that the responses of vagal afferents to intra-intestinal glucose is modulated by intravenous glucose (Mei, 1978) suggesting that circulating glucose may also modulate the activity and responsiveness of vagal sensory neurons.

Despite being contained within the relatively tough capsule of the nodose ganglion, vagal sensory neurons appear to be accessible to circulating factors (Lacolley et al., 2006a,b). While glucose is a universal fuel for neurons, some neurons possess the additional ability of using variations in extracellular glucose levels as a means of altering their excitability (Adachi et al., 1995; Levin et al., 2001; Kang et al., 2006). A subpopulation of vagal sensory neurons appears to display this sensitivity and are either excited or inhibited by elevations in glucose levels (Grabauskas et al., 2010). Further, the response to glucose appears to be related to the visceral organ that the vagal sensory neurons innervate; afferent neurons projecting to the stomach are more likely to exhibit excitatory responses to elevations in glucose levels while those that innervate the portal vein were more likely to be inhibitory in response to an increase in glucose, suggesting that the effects of glucose on vagal sensory transmission are specialized relative to the visceral information they transmit (Grabauskas et al., 2010). As with other neurons excited by elevations in glucose levels, this activation appears to involve the closure of an ATP-sensitive potassium channel (Dunn-Meynell et al., 1998; Ferreira et al., 2001; Raupach and Ballanyi, 2004; Balfour and Trapp, 2007; Grabauskas et al., 2010) while inhibition of vagal sensory neurons by glucose appears to involve an ATP-insensitive potassium channel although the ionic current involved remains to be elucidated (Grabauskas et al., 2010).

In addition to direct actions upon vagal afferent neurons, glucose also exerts indirect actions via modulation of neurotransmitter receptor density on the neuronal surface. In particular, we have demonstrated recently that glucose induces trafficking of 5-HT_3_ receptors in GI vagal afferent neurons; following an increase in glucose levels, 5-HT_3_ receptors are trafficked to the neuronal membrane (Babic et al., 2012). In contrast, a decrease in glucose levels results in receptor internalization (Babic et al., 2012). The functional consequence of this glucose-induced receptor trafficking is an increase or decrease in the inward current induced by 5-HT in response to an elevation or reduction of extracellular glucose level (Babic et al., 2012). Importantly, this glucose-induced modulation of 5-HT_3_ receptor function occurs rapidly (within minutes) suggesting that, in addition to inducing the release of 5-HT from enterochromaffin cells, glucose may also increase the ability of GI vagal afferent neurons to respond to released 5-HT. In this regard, it is also notable that, following the glucose-induced release of GI neurohormones and their activation of vagal afferent terminals within the intestine, these neurohormones also enter the circulation and gain access to vagal afferent neurons; circulating levels of platelet free 5-HT increase approximately 3-fold after meal ingestion (Houghton et al., 2003). Thus, the glucose-induced modulation of 5-HT_3_ receptor density and function on vagal afferent neurons appears to be a means by which sensory signaling from the GI tract can be amplified or prolonged.

## Effects of glucose on central vagal neurocircuits

In addition to modulating the activity and functions of vagal sensory neurons and peripheral terminals, glucose also modulates the release of neurotransmitter from the central terminals of vagal sensory neurons; increasing the extracellular glucose concentration increased action potential-dependent and -independent glutamate release onto second order NTS neurons, while decreasing extracellular glucose levels inhibited glutamate release (Wan and Browning, 2008a). Further studies demonstrated that, as with vagal sensory somata, glucose induces the trafficking of 5-HT3 receptors to the membrane of vagal sensory nerve terminals, the activation of which increases glutamate release (Wan and Browning, 2008b). 5-HT_3_ receptors on vagal afferent terminals appear to be activated tonically; in fact, the 5-HT_3_ receptor antagonist, ondansetron, decreases action potential dependent and independent synaptic transmission to second order NTS neurons implying an ongoing activation of these receptors (Wan and Browning, 2008b). The NTS receives a dense serotonergic input from other brainstem nuclei, most prominently the medullary raphe nuclei (Steinbusch, 1981; Steinbusch and Nieuwenhuys, 1981; Thor and Helke, 1989) although vagal afferent neurons themselves contain 5-HT (Nosjean et al., 1990; Sykes et al., 1994).

Early studies using extracellular recording techniques showed that glucose is able to modulate the activity of subpopulations of neurons within the brainstem. Hepatic vagal afferent fibers that showed a decrease in activity in response to increased glucose exposure, for example, innervate NTS neurons that are also inhibited by local application of glucose (Adachi et al., 1984). Other NTS neurons, in contrast, increased their activity in response to elevated glucose levels (Adachi et al., 1995; Yettefti et al., 1995, 1997; Dallaporta et al., 2000); as described earlier for vagal sensory neurons, as well as for other glucose-sensitive central neurons (Dunn-Meynell et al., 1998; Levin et al., 2001), this increase in neuronal activity in response to increased extracellular glucose levels appear to involve ATP-sensitive potassium channels (Dallaporta et al., 2000; Balfour et al., 2006; Balfour and Trapp, 2007). The mechanism responsible for glucose-induced neuronal inhibition awaits further study, although modulation of chloride conductances may be involved (Balfour and Trapp, 2007).

The ability of glucose to modulate the activity of DMV neurons is more controversial, however. An earlier study using extracellular recording techniques suggested that a small subpopulation of DMV neurons that project via the ventral, or anterior, gastric branch increased or decreased their activity in response to topical glucose administration (Kobashi and Adachi, 1994; Adachi et al., 1995). Other studies using whole cell patch clamp recording techniques confirmed the presence of ATP-sensitive potassium channels on DMV neurons (Trapp et al., 1994; Karschin et al., 1998; Ferreira et al., 2001; Kulik et al., 2002; Raupach and Ballanyi, 2004; Balfour et al., 2006; Balfour and Trapp, 2007; Blake and Smith, 2012) suggesting their activity may be modulated by extracellular glucose levels. In contrast, later studies failed to observe any direct effects of glucose on DMV neuronal activity but, rather, demonstrated indirect effects via modulation of synaptic inputs presumably from NTS neurons (Ferreira et al., 2001). In part, these studies highlight the potential difficulties in separating direct from indirect effects when recording from neurons in brain slice preparations and the additional care required when examining ATP-sensitive potassium channels in neurons (to prevent unintentional channel closure via ATP supplied in the intracellular patch pipette solution).

**Figure 1:**
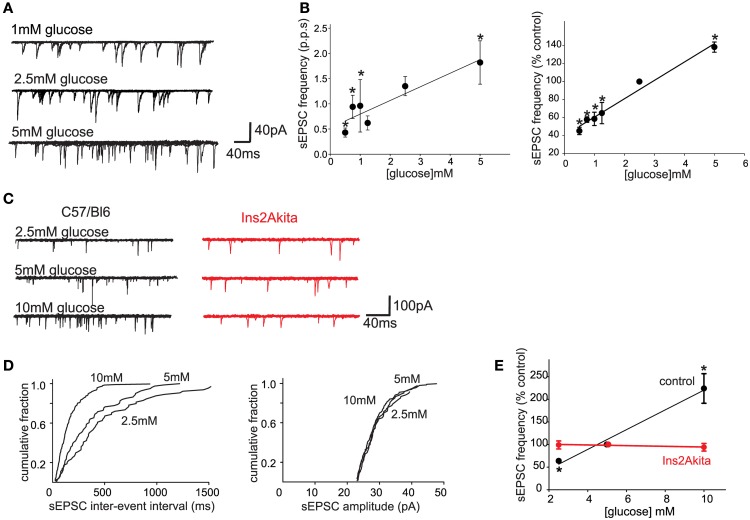
**Physiological extracellular glucose levels modulate glutamate release from the central terminals of vagal afferent neurons, an effect that is lost in diabetes. (A)** Previously, we demonstrated that glucose acted presynaptically to increase the release of glutamate from the central terminals of vagal afferents (Wan and Browning, 2008b). To determine whether the release of glutamate is modulated by more physiological levels of glucose, whole cell patch clamp recordings were made from 27 neurons of the NTS subnucleus centralis (cNTS); neurons were voltage clamped at −60 mV and the effects of exposure to different physiological concentrations of extracellular glucose on spontaneous excitatory postsynaptic currents (sEPSCs) were recorded. A neuron was considered responsive if glucose altered either frequency or amplitude of sEPSCs by at least 20%. The proportion of neurons responding to a decrease in extracellular glucose concentration with a decrease in sEPSC frequency were as follows: from 2.5 to 0.5 mM, 4/6 neurons responded; from 0.5 to 0.75 mM, 6/7 neurons responded; from 2.5 to 1 mM, 5/6 neurons responded; from 2.5 to 1.25 mM, 4/6 neurons responded. In contrast, increasing extracellular glucose concentration from 2.5 to 5 mM increased sEPSC frequency in 6/7 neurons tested. Even within this narrow, presumably more physiological, range of glucose concentrations (0.5–5 mM), the frequency, but not amplitude, of sEPSCs was modulated by extracellular glucose concentration. **(B)** Summary graphics illustrating the relationship between extracellular glucose level and frequency of sEPSCs in cNTS neurons in rats. When expressed both as absolute frequency (pulse per second; p.p.ps; left) and as a percentage of sEPSC frequency at control glucose level (2.5 mM; right), the frequency of sEPSCs was dependent upon extracellular glucose concentration in a linear manner. ^*^P < 0.05 vs. 2.5 mM glucose. These results suggest that the ability of glucose to modulate glutamate release from the central terminals of vagal afferents occurs across a wide range of conditions both physiological, as well as pathophysiological. The rapid alteration in glutamate transmission in response to extracellular glucose levels further suggests that glucose may set a background “tone” or ongoing level of transmission from the central terminals of gastrointestinal vagal afferents that can be up- or down-regulated in an ongoing, and rapidly reversible, manner. **(C)** To determine whether the sensitivity of vagal afferent terminals to glucose (Wan and Browning, 2008b) is (a) also present in the mouse and (b) altered during pathophysiological conditions such as diabetes, whole cell patch clamp recordings were made from NTS subnucleus centralis (cNTS) neurons (*n* = 9) in control (C57/Bl6) mice as well as from neurons (*n* = 8) in a mouse model of spontaneously developing Type 1 diabetes (Ins2Akita mice). As in the rat, the frequency of sEPSCs in cNTS neurons was dependent upon the extracellular glucose level in C57/Bl6 mice (left). In detail, in 5/6 neurons tested, decreasing the extracellular glucose concentration from 5 to 2.5 mM decreased sEPSC frequency; in contrast, in 6/7 neurons tested, increasing the extracellular glucose concentration from 5 to 10 mM increased sEPSC frequency. In no instance was any effect on sEPCS amplitude observed (113 ± 10% of control amplitude in 2.5 mM glucose and 100 ± 6% of control amplitude in 10 mM glucose; *P* > 0.05 in each case). In contrast, the ability of glucose to modulate the frequency of sEPSCs was lost in neurons from Ins2Akita mice (right). In detail, 4/5 neurons tested, decreasing the extracellular glucose concentration from 5 to 2.5 mM had no effect on sEPSC frequency while increasing extracellular glucose concentration from 5 to 10 mM had no effect on sEPSC frequency in any of the 5 neurons tested. **(D)** Computer generated graphics from the same control C57/Bl6 neuron as above showing that glucose increases the frequency (left) but not amplitude (right) of sEPSCs suggesting that, as in the rat, glucose acts at presynaptic sites to modulate glutamate release. **(E)** Summary graphic illustrating the relationship between extracellular glucose level and frequency of sEPSCs in cNTS neurons from control C57/Bl6 and diabetic Ins2Akita mice. When expressed a percentage of frequency at control glucose level (5 mM glucose), the ability of glucose to modulate the frequency of sEPSCs was lost in diabetes. ^*^P < 0.05 vs. control frequency. These results suggest that the ability of glucose to modulate glutamatergic transmission from the central terminals of vagal afferents may be a more generalized phenomenon that occurs across species. The results further suggest that the glucose-dependent modulation of central vagal neurocircuits is compromised by chronic hyperglycemia. The timing of this loss in responsiveness, as well as its cause-or-effect response to the development of diabetes, may provide valuable insights into the effects of acute vs. chronic hyperglycemia on autonomic neurocircuitry and the consequence effects on gastrointestinal homeostatic regulation including the gastric dysmotility and delayed gastric emptying observed during hyperglycemia/diabetes.

One important caveat to the studies investigating the role of glucose within the brainstem is the concentration or dose of extracellular glucose used in most studies, which almost certainly exceeds physiological levels. Extracellular glucose levels in most CNS regions are assumed to be 15–20% of peripheral levels; hypothalamic glucose levels certainly vary in concert with blood glucose levels, but they do so within a very narrow range (~0.25–1.0 mM; Dunn-Meynell et al., 2009) although other studies have measured cortical glucose levels between 0.2 and 4.5 mM (Silver and Erecinska, 1994). It should be borne in mind, however, that brainstem vagal neurons may be exposed to higher glucose levels than many other central nuclei since they are essentially circumventricular organs with a leaky blood-brain barrier and fenestrated capillaries (Cottrell and Ferguson, 2004). Regardless, our laboratory has demonstrated recently that, even at similarly low (0.5–5 mM, presumably more physiological) levels, glucose modulates glutamate release from the central terminals of vagal afferent neurons onto second order NTS neurons (Figure 1; Browning, unpublished data). This would suggest that the central terminals of some GI vagal sensory neurons may function as glucose sensors, in the sense that extracellular glucose levels regulate neurotransmitter release *in a linear* fashion across both physiological and pathophysiological ranges (Wan and Browning, 2008a).

## Alterations in actions of glucose on vagally-mediated gastrointestinal reflexes during pathophysiological states

As described earlier, GI functions including gastric motility and emptying are modulated by physiological alterations in blood glucose levels (Rayner et al., 2001). It is hardly surprising, therefore, that pathophysiological alterations in glucose levels result in profound disruption of GI functions. Although gastric hyperactivity has been observed in some rodent models of hyperglycemia and is experienced by some patients with diabetes, a significant proportion of animal models as well as patients exhibit diabetic gastroparesis, defined as delayed gastric emptying accompanied by other upper GI symptoms such as early satiety, fullness, abdominal pain, bloating, and nausea (Horowitz et al., 2002; Chaikomin et al., 2006). The severity of diabetic gastroparesis can vary widely from symptoms of mild discomfort up to impaired glycemic control, electrolyte imbalance, and malnutrition (Horowitz et al., 2002; Chaikomin et al., 2006). Despite the considerable healthcare and social costs associated with this disease, the pathophysiology of diabetic gastroparesis remains to be elucidated fully. That both Type 1 and Type 2 diabetic patients experience gastroparesis symptoms suggests that hyperglycemia, or dysregulated glycemic control *per se*, may play an important role in symptom development, although insulin itself certainly modulates the activity of central vagal motoneurons (Blake and Smith, 2012) and induces vagally-mediated increases in gastric motility (Krowicki et al., 1998).

**Figure 2:**
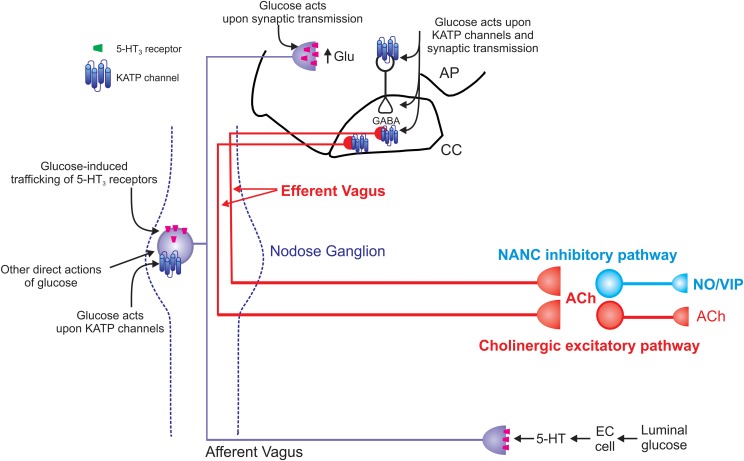
**Schematic representation of the effects of glucose on vago-vagal reflex control of the stomach.** Glucose within the intestine induces the release of serotonin from enteroendocrine (EC) cells (Zhu et al., 2001; Freeman et al., 2006). The released 5-HT acts upon 5-HT3 receptors present on the peripheral terminals of vagal afferent neurons to cause their excitation (Hillsley et al., 1998; Raybould et al., 2003; Grundy, 2006); this peripheral signal is relayed centrally via the afferent vagus nerve. Once absorbed into the circulation, glucose is also able to act directly upon vagal afferent neurons within the nodose ganglion. Glucose induces neuronal excitation via actions upon ATP-sensitive potassium channels (KATP channels; Grabauskas et al., 2010) as well as causing neuronal inhibition via an as yet unidentified mechanism (Grabauskas et al., 2010). Glucose also rapidly and reversibly traffics 5-HT3 receptors to and from the membrane of gastrointestinal vagal afferent neurons (Babic et al., 2012). Since circulating platelet-free 5-HT levels increase following ingestion of a meal (Houghton et al., 2003), this provides a means by which glucose is able to modulate its own “perception” and amplify or prolong vagal afferent signaling. The central terminals of vagal afferent neurons enter the brainstem via the tractus solitarius and terminate on NTS neurons using predominantly glutamate as a neurotransmitter (Andresen and Yang, 1990; Andresen and Kunze, 1994). Glucose is also able to modulate the release of glutamate from the central terminals of vagal afferents by actions that involve 5-HT3 receptors (Wan and Browning, 2008a,b). Glucose can also activate NTS neurons via actions on KATP channels and increase synaptic transmission to gastric-projecting DMV neurons (Adachi et al., 1984, 1995; Ferreira et al., 2001). Glucose can also modulate the activity of DMV neurons directly (Trapp et al., 1994; Karschin et al., 1998; Balfour et al., 2006; Balfour and Trapp, 2007). The result of these central and peripheral actions of glucose is gastric relaxation and delayed gastric emptying (Schvarcz et al., 1997; Raymer et al., 2000; Rayner et al., 2001). The vagal efferent pathway involved in this gastric relaxation and delayed gastric emptying is still controversial. Peripheral actions of glucose appear to involve activation of a non-adrenergic, non-cholinergic pathway (Zhou et al., 2008) whereas central glucose appears to involve inhibition of the tonically active cholinergic pathway (Ferreira et al., 2001; Shi et al., 2003, 2005).

The GI dysfunctions induced by either Type 1 or Type 2 diabetes may occur through actions at multiple sites (Figure 2). A loss of enteric neurons neurons, particularly inhibitory neurons (nitric oxide synthase-, vasoactive intestinal peptide-, neuropeptide Y- and galanin-immunoreactive) has been reported in rodent models of both Type 1 and Type 2 diabetes during the early stages of the disease [reviewed in Chandrasekharan and Srinivasan (2007)] which may contribute to the observed disordered motility patterns and decreased NANC-dependent muscle relaxations (Jenkinson and Reid, 2000; Yoneda et al., 2001; Demedts et al., 2013). Loss of Inhibitory Cells of Cajal (ICC) has also been reported in both Type 1 and Type 2 diabetes (Ordog et al., 2000; He et al., 2001; Iwasaki et al., 2006; Forrest et al., 2008; Wang et al., 2009; Grover et al., 2011) suggesting this may be another important means by which persistant hyperglycemia dysregulates GI motility although reduced levels of insulin and insulin signaling, rather than hyperglycemia *per se*, has also been shown to be involved in ICC depletion (Horvath et al., 2005).

Glucose sensing within enteroendocrine cells is also disrupted in diabetes (Lee et al., 2012) as are both the basal expression and function of intestinal sodium-glucose transporters (Bihler and Freund, 1975; Morton and Hanson, 1984; Dyer et al., 2002; Bhutta et al., 2013) suggesting that the increased absorption of glucose further amplifies the disrupted and dysregulated responses of GI neurocircuits to glucose. It is hardly surprising, therefore, that altered vagal sensory and motor fiber functions have been reported in both humans (Tougas et al., 1992) and rodent models of diabetes (Yagihashi and Sima, 1986; Lee et al., 2001, 2012; Regalia et al., 2002). While frank autonomic neuropathy almost certainly contributes to the altered vagal sensory and motor functions observed in chronic diabetes, the actions of acute hyperglycemia to modulate vagal afferent and efferent functions (MacGregor et al., 1976; Shi et al., 2003; Takahashi et al., 2003; Zhou et al., 2008) suggests that poor glycemic control *per se* also negatively impacts vagal reflex functions.

While diabetes is most often considered a peripheral metabolic disease, an increasing body of evidence indicates a significant involvement of the central nervous system, including vagal neurocircuits within the hindbrain, in its development and functional outcomes. In a mouse model of spontaneously developing Type 1 diabetes, the Ins2(Akita) mouse, the ability of glucose to modulate synaptic transmission to second order NTS neurons is lost (see Figures 1C,D; Browning, unpublished data) suggesting an impairment of glucose sensitivity within vagal sensory neurocircuits. Recent studies have demonstrated that even short time periods of glycemic dysregulation result in significant modulation of synaptic transmission within vagal neurocircuits (Zsombok et al., 2011). This would suggest that the vagal control of GI functions may by disrupted even in the early stages of glycemic dysregulation, rather than as a consequence of autonomic neuropathy. In this regard, preliminary evidence that even short periods of exposure to a high fat diet disrupts the glucose-induced trafficking of 5-HT_3_ receptors on GI vagal sensory neurons, well in advance of the development of obesity or hyperglycemia (Troy and Browning, 2013), raises the possibility that altered glucose signaling within vagal neurocircuits may precede, and even contribute to, disease development.

## Future directions

Glucose sensing within autonomic neurocircuits is critical for the effective integration and regulation of a variety of physiological homeostatic functions involved in the optimal regulation of blood glucose levels, including the co-ordination of vagally-mediated reflexes regulating GI functions (e.g., gastric motility and emptying, nutrient absorption and satiety signaling). Glucose regulates GI functions via actions at multiple sites of action, from modulating the activity of enteric neurons, endocrine cells and glucose transporters within the intestine, to regulating the activity and responsiveness of the peripheral terminals, cell bodies and central terminals of vagal sensory neurons, to modifying both the activity and synaptic responsiveness of NTS and DMV neurons. Unsurprisingly, significant impairment in GI functions results occurs in pathophysiological states where glucose levels are dysregulated, such as diabetes. A substantial obstacle to the development of new therapies to modify the disease, rather than treat the symptoms, are the gaps in our understanding of the mechanisms by which glucose modulates GI functions, particularly vagally-mediated responses. Vagal afferent and efferent fibers represent a much more readily available target for new therapies and a more complete understanding of disease-related plasticity within these neurocircuits may open new avenues and targets for research.

It will be of particular interest to elucidate the reversibility of hyperglycemia- and diabetes-induced vagal dysregulation—is there a period of exposure to hyperglycemia beyond which vagal neural damage is irreversible or is the neurocircuitry sufficiently plastic to recover with subsequent tight glycemic control? In this regard, the recent demonstration that the diet-induced obesity associated decrease in excitability and responsiveness of vagal motoneurons is reversed completely by Roux-en-Y gastric bypass surgery (Browning et al., 2013) suggests that vagal neurocircuits remain open to adaptation and that long-term dysregulation of their activity does not necessarily result in permanent and irrecoverable damage. The rapid remission of Type 2 diabetes following bariatric surgery, far in advance of weight loss, raises questions as to its mechanism of action and the degree to which recovery of glycemic regulation is related to the recovery of vagal afferent and efferent homeostatic control.

### Conflict of interest statement

The author declares that the research was conducted in the absence of any commercial or financial relationships that could be construed as a potential conflict of interest.
